# A modified method for isolation of bladder cancer stem cells from a MB49 murine cell line

**DOI:** 10.1186/1471-2490-13-57

**Published:** 2013-11-04

**Authors:** Yong-tong Zhu, Cheng-yong Lei, Yang Luo, Na Liu, Cheng-wu He, Wei Chen, Fei Li, Yong-jian Deng, Wan-long Tan

**Affiliations:** 1 Department of Urology, Nanfang Hospital, Southern Medical University, Guangzhou, Guangdong 510515, P.R. China; 2 Department of Gynecology, Zhujiang Hospital, Southern Medical University, Guangzhou, Guangdong 510515, P.R. China; 3 Department of Pathology, Southern Medical University, Guangzhou, Guangdong 510515, P.R. China

**Keywords:** Bladder cancer, MB49 cell line, Cancer stem cells, Proliferation, Chemotherapy

## Abstract

**Background:**

The vaccine was efficiently effective against bladder cancer in earlier studies. However, a part of the mouse bladder tumour regrew due to regression after a period of time as the cancer stem cells could not be eliminated. In this study, we showed a modified method for the isolation of MB49 bladder cancer stem cells (MCSCs).

**Methods:**

Through a comparison of different serum-free culture mediums (SFM), MCSCs were isolated by a combination of the limited dilution method and the optimal SFM method. The characterizations of MCSCs were verified by the fluorescence activated cell sorting, the quantitative polymerase chain reaction, the western blotting, the cell proliferation assay, the soft agar assay, the transwell assay, the resistance to chemotherapy assay and the tumor xenograft formation assay.

**Results:**

The optimal SFM contained a RPMI1640+ epidermal growth factor (20 ng/ml), a basic fibroblast growth factor (20 ng/ml), a leukemia inhibitory factor (20 ng/ml), a B-27 serum-free supplement (20 μl/ml), and a bovine serum albumin (4 μg/ml). MCSCs possessed the high expression of cancer stem cell markers (CD133, CD44, OCT4, NANOG, and ABCG2) and the ability of differentiation. In functional comparisons, MCSCs had higher proliferative abilities, lower susceptibility to chemotherapy, greater migration in vitro, and stronger tumorigenic abilities in vivo.

**Conclusion:**

MCSCs displayed specific cancer stem cells properties. Our study showed MCSCs were isolated successfully with a modified method using a combination of limited dilution and SFM methods.

## Background

The MB49 bladder cancer cell vaccine induced a specific antitumor immunity and was efficiently effective against metastatic bladder cancer in our earlier studies [1-3]. However, we also found that a part of the mouse bladder tumor regrew after experiencing regression for a period of time because the cancer stem cells (CSCs), or cancer-initiating cells, could not be eliminated. Recent findings supported the notion that relapses of solid tumors may be attributed to the inability of traditional chemotherapies and radiotherapies to eradicate CSCs [4]. Our former bladder cancer vaccine was not the CSC vaccine, which was unable to induce specific immunities responsible for CSCs.

Limited dilution assays were first used to establish CD133^+^ single cell-derived progenies of colorectal cancer in 2010 [5]. The serum-free culture medium (SFM) method had been used to isolate CSCs from tumors, but it was limited due to the lack of purity in the CSCs [6]. As we known, the combination of the limited dilution method and SFM method has not been used to isolate the CSCs, which could improve the purity of cell sorting. Cancer stem cells from a MB49 bladder cancer cell line (MCSCs) had not been demonstrated before, and the isolation of MCSCs would provide a model for the development of bladder cancer vaccine research. We provide a modified method here by combining the limited dilution and SFM methods to isolate MCSCs.

## Methods

### Optimal SFM for MCSCs

The mediums reported previously to support the expansion of CSCs were different [7,8]. Furthermore, the defined SFM formulations reported in previous literature have not been able to support the MCSCs. Based on these considerations, a sequential approach was taken to identify defined SFMs for the successful isolation and expansion of MCSCs.

The culture media and recombinant growth factors tested are shown in Table 1. The epidermal growth factor (EGF), fibroblast growth factor basic (FGF-b), leukemia inhibitory factor (LIF), and the B-27 serum-free supplement (B27) have previously supported the expansion of different kinds of CSCs [9,10]. The most important serum substitute is bovine serum albumin (BSA), and the optimized concentration of BSA is 0.4% [11]. The different combinations shown in Table 2, e.g. No. 5 are EGF + B27 + BSA + RPMI1640.

**Table 1:** Cell culture medium and supplements

**Reagent**	**Suppliers**	**Concentration**
EGF	Peprotech, Rocky Hill, NJ, USA	20 ng/ml
FGF-b	Peprotech	20 ng/ml
LIF	eBioscience, San Diego, CA, USA	20 ng/ml
B27	Invitrogen, Grand Island, NY, USA	20 μl/ml
BSA	Thermo Scientific HyClone, Logan, UT, USA	4 mg/ml
RPMI1640	Thermo Scientific HyClone	1×
DMEM/F12	Thermo Scientific HyClone	1×

**Table 2:** Combination of medium and supplements

**No.**	**1**	**2**	**3**	**4**	**5**	**6**	**7**	**8**	**9**	**10**	**11**	**12**	**13**	**14**	**15**	**16**
EGF			√		√			√	√		√		√	√	√	√
FGF-b				√			√		√	√	√	√		√	√	√
LIF		√				√	√	√			√	√	√		√	√
B27	√				√	√				√		√	√	√	√	√
BSA	√	√	√	√	√	√	√	√	√	√	√	√	√	√	√	√
RPMI 1640	√	√	√	√	√	√	√	√	√	√	√	√	√	√		√
DMEM/F12															√	

#### Cell culture for the MB49 cell line

The murine bladder cancer cell line, MB49, was a gift from Dr. I. C. Summerhayes from the Lahey Clinic in Burlington, Massachusetts [1-3]. MB49 cells were cultured in RPMI1640 that contained 10% fetal bovine serum (FBS, Thermo Scientific HyClone, Logan, Utah) at 37°C in a 5% CO_2_ humidified incubator.

#### Comparison of culture medium and supplements

The accutase-enzyme cell detachment medium (Accutase, eBioscience, San Diego, California) was used for digestion. Then the different culture mediums were added to dilute the Accutase in order to stop digestion. Finally, the MB49 cells were dissociated into the single cell suspension and seeded at a density of 2 × 10^3^ cells per well as shown in Table 2. There were various culture mediums in 96-well plates with ultra low attachment surface (Corning Life Sciences, Union City, California). Cells were incubated for a subsequent 5 days. Then 10 μl of the Cell Counting Kit-8 reagent (CCK-8, Dojindo Molecular Technologies, Kumamoto, Japan) were added at a fixed time in 1, 2, 3, 4, and 5 days. After a 4 hour incubation, the absorbance value was measured at 450 nm using an EnSpire 2300 multilabel reader (PerkinElmer, Singapore).

After 7 days, the colonies with a diameter greater than 50 μm were counted with an inverted microscope (Nikon, Japan), and the cell morphologies in various culture mediums were recorded with a camera (Nikon, Japan).

### Establishment of MCSCs

#### Limited dilution method

The MB49 cells were digested with Accutase. They were then counted and diluted tenfold at a limited 3–4 times to form a density of 5 cells per milliliter in an optimal SFM with plated 200 μL in a 96-well plate. Finally, single cells were marked and observed every day.

#### Passage culture

The Passage 1 single cells were cultured in the optimal SFM, and we removed the supernatant and supplemented fresh SFM every 5–7 days. By the 30th day, the single cells had grown to single-cell spheres so large that they were visible. They were digested with Accutase, dispersed mechanically, and plated in a 24-well plate with an ultra low attachment surface (Corning Life Sciences) before forming passage 2 cells.

The Passage 2 cells were cultured with freshly changed SFM every 3–4 days. By the 15th day, most of the cells had grown to spheres large enough to view. Then the spheres were collected, centrifuged for 5 minutes at 800 rpm, digested with Accutase, dispersed mechanically, and plated in a 6-well plate with an ultra low attachment surface (Corning Life Sciences) before forming passage 3 cells. The cells had expanded to a T25 culture flask (Corning Life Sciences) through multiple passages using the same protocol.

### Characterizations of MCSCs

#### Expression of MCSCs markers

##### Fluorescence activated cell sorting (FACS)

The MB49 cells and MCSCs were harvested respectively. They were dissociated at a density of 1 × 10^4^ cells in a 100 μl autoMACS running buffer (Miltenyi Biotec, Bergisch Gladbach, Germany), labeled with 20 μl PE mouse anti-prominin-1 (Miltenyi Biotec) and FITC mouse antiCD44 (Miltenyi Biotec), incubated for 20 minutes at 4°C, and washed twice with phosphate buffered saline (PBS). To set the background fluorescence levels, we used the PE rat IgG1 *κ* isotype control (eBioscience) and the TITC rat IgG2b *κ* isotype control (eBioscience) as the negative control. The ratio of CD44^+^CD133^+^ cells was evaluated using a BD FACSAria cell sorter (Becton-Dickinson, San Jose, California).

##### Quantitative polymerase chain reaction (qPCR)

The total RNAs extracted were isolated by using the Arcturus PicoPure RNA isolation kit (Applied Biosciences, Carlsbad, New Mexico). The RNA quality was verified by the Bioanalyzer RNA Pico Chip (Agilent Technologies, Santa Clara, California). The two micrograms of total RNA were reverse transcribed with Superscript III (Invitrogen, Grand Island, New York) to synthesize the first-strand cDNA. The cDNA was amplified with SYBR green PCR master mix (Bio-Rad, Hercules, California) on a 7500 real time PCR system (AB Applied Biosystems, Singapore). The cycling conditions were 95°C for 10 s (denaturation) and 60°C for 60 s (annealing and extension). The primer sequences are listed in Table 3. Normalization and fold changes were calculated using the ∆∆C_t_ method[12]. The gene expression of GAPDH was used as a negative control.

**Table 3:** Primers of selected genes

**Gene name**	**Primers (forward/reverse)**	**Base pairs of product**
CD133	F: 5’-CGGGATCCGAAAAACTGATCTGT-3’	615 bp
	R: 5’-CCGCTCGAGTTACCTAGTTACTCTCTCC-3’	
CD44	F: 5’-CCCTGCTACCAGAGACCAAGAC-3’	401 bp
	R; 5’-GCAGGTTCCTTGTCTCATCAGC-3’	
NANOG	F: 5’-CAGCTGTGTGTACTCAATGATAGATTT-3’	179 bp
	R: 5’-ACACCATTGCTATTCTTCGGCCAGTTG-3’	
OCT4	F: 5’-TCAGCCAAACGACCATCTGC-3’	205 bp
	R: 5’TTCTCCAGGTTGCCTCTCAC-3’	
GAPDH	F: 5’-CCATGGAGAAGGCTGGGG-3’	198 bp
	R: 5’-CAAAGTTGTCATCCATGACC-3’	

##### Western blotting (WB)

Equal amounts of the protein samples extracted were separated with 10% sodiumdodecyl sulfate -polyacrylamide gel and transferred to polyvinylidene difluoride membranes (Millipore, Billerica, Massachusetts) electrophoretically. Filters were blocked in the PBS with 5% skim milk and incubated overnight at 4°C with the primary antibody anti-OCT4 (Abcam, Cambridge, Massachusetts), anti-NANOG (Abcam), anti-ABCG2 (Abcam), and anti-β-actin antibody (Abcam). The filters were then incubated with conjugated anti-mouse secondary antibodies (Abcam)[13]. The protein bands were detected by Fluor Chem FC2 (Alpha Innotech, San Leandro, California) and analyzed by Image Lab software.

#### Differentiation

The MCSCs were collected, dissociated into single cells, and cultured in RPMI1640 supplemented with 10% FBS to induce cell differentiation. Meanwhile, the MCSC spheres were cultured by the same method.

#### Functional comparison

##### Cell proliferation assay

The cells were plated at a number of 1 × 10^3^ in a 96-well plate and incubated for 1, 2, 3, 4, 5, and 6 days respectively. We then added 10 μl CCK-8, the samples were incubated for 4 hours, and the absorbance values were measured as before.

##### Soft agar assay

The cells were resuspended at a density of 1 × 10^4^/ml with a bottom of 0.66% agar (Beyotime, Jiangsu, China) while the medium was supplemented with 10% FBS and layered on the top was a 1.32% agar supplemented with 20% FBS on 6-well plates respectively[13]. The plates were incubated for three weeks, and then the colonies with diameters greater than 50 μm were counted.

##### Migration abilities in vitro

The cells were seeded at a number of 1 × 10^4^ in 0.25 ml of pure RPMI1640 on a 6.5-mm pore-size polycarbonate membrane chamber inserted in a transwell apparatus (Costar, Cambridge, Massachusetts). 0.75 ml of the RPMI1640 medium that contained 10% FBS was added to the lower chamber. Then the cells were incubated for 24 hours. The cells that had migrated to the bottom surface of the insert were fixed in paraformaldehyde for 20 minutes, stained in giemsa for 15 minutes, rinsed in PBS, and inspected via inverted microscopy.

##### Resistance to chemotherapy abilities

The cells were seeded at a number of 1 × 10^4^ in a 96-well plate. After 24 hours, the chemotherapeutic agents mitomycin (Sigma-Aldrich, St. Louis, Missouri), cisplatin (Sigma-Aldrich), paclitaxel (Sigma-Aldrich), and doxorubicin (Sigma-Aldrich) were added with different concentrations (Table 4). The cells were treated for a subsequent 96 hours. Therefore, 10 μl of CCK-8 were added to each well, and after 4 hours of incubation, the absorbance values were measured. The cell viability that corresponded to each drug treatment was expressed as the percentage of absorbance values of the treated wells related to the untreated control wells [12].

**Table 4:** Concentrations of chemotherapeutic agents

**Agents**	**Concentrations**			
Paclitaxel	10 nM	100 nM	1 μM	10 μM
Doxorubicin	10 nM	100 nM	1 μM	10 μM
Cisplatin	5 μM	10 μM	15 μM	20 μM
Mitomycin	10 μM	80 μM	640 μM	5.12 mM

##### Tumorigenic abilities in vivo

All of the experimental procedures with animals used in the present study have been given prior approval by the Ethics Committee of Southern Medical University under Contract 2011016. 4-week-old immune deficient nude mice (Center of Experimental Animals, Southern Medical University, Guangzhou, China) were maintained and treated under specific, pathogen-free conditions. The cells were injected with gradient concentration subcutaneously into the nude mice, at a number from 1 × 10^2^ to 1 × 10^4^ in MCSCs and from 1 × 10^4^ to 1 × 10^6^ in MB49 cells. The tumor xenograft formation was observed every week. At the end of eight weeks, the mice were sacrificed by cervical dislocation, the tumor engrafts were removed, and the volume of tumors was measured by using the formula d^2^ × D/2, where d and D were the shortest and the longest diameters respectively [14].

### Statistical analysis

SPSS19.0 software was used for the statistical evaluations. All of the data was expressed as the mean ± standard deviation and analyzed using one-way ANOVA. P < 0.05 was considered statistically significant.

## Results

### Comparison of culture medium and supplements for MB49 cell culture

**Figure 1:**
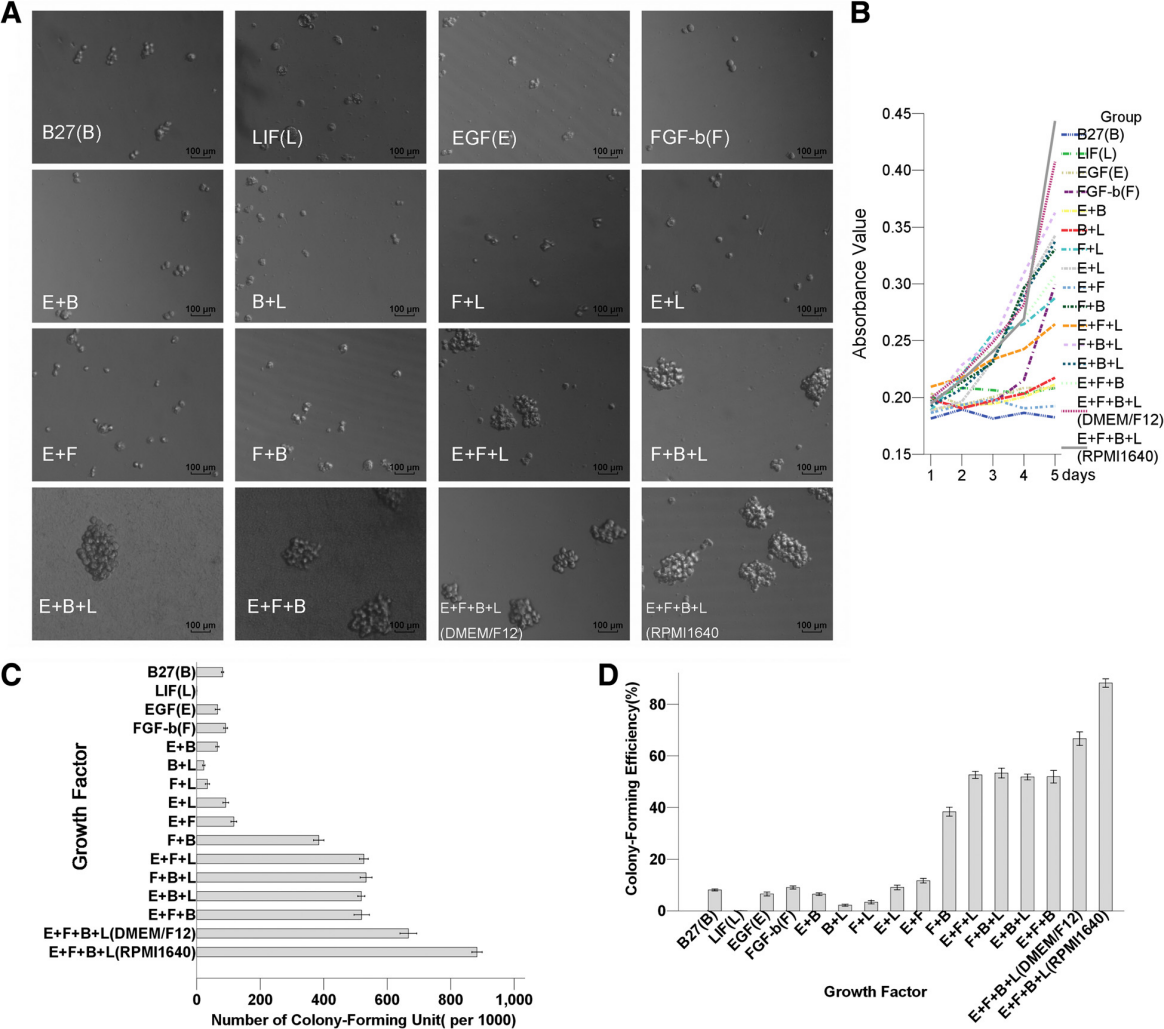
**Comparison of culture mediums and supplements for MB49 cell cultures.** The combination for an optimal SFM is RPMI1640+ EGF(E) + FGF-b(F) + LIF(L) + B27(B) + BSA. **(A)** SFM with the combination of all four growth factors E + F + L + B together provides a robust synergistic effect on MB49 cell morphology. The medium containing just one or two growth factors displays small sphere morphology. **(B)** The cell proliferation curve shows that the combination of E + F + L + B contains the highest absorbance value after using the Cell Counting Kit-8(CCK-8). **(C)** The largest numbers of colony-forming units is the combination of E + F + L + B. **(D)** The highest sphere-formation efficiency is the combination of E + F + L + B.

The SFM with the combination of all four growth factors (EGF, FGF-b, LIF, and B27) together provided a robust synergistic effect on cell proliferation. The medium contained just one or two growth factors and displayed small sphere morphology (Figure 1a). The cell proliferation curve showed that the combination of EGF, FGF-b, LIF, and B27 contained the highest absorbance value using CCK-8 (Figure 1b). The numbers of colony-forming units and colony-forming efficiencies showed the same trend (Figure 1cd). The cells grown in DMEM/F12 displayed a similar colony-forming potential compared to those grown in RPMI1640. The combination of the optimal SFM was RPMI1640 + EGF (20 ng/ml) + FGF-b(20 ng/ml) + LIF(20 ng/ml) + B27(20 μl/ml) + BSA(4 μg/ml).

### Establishment of MCSCs in SFM

**Figure 2:**
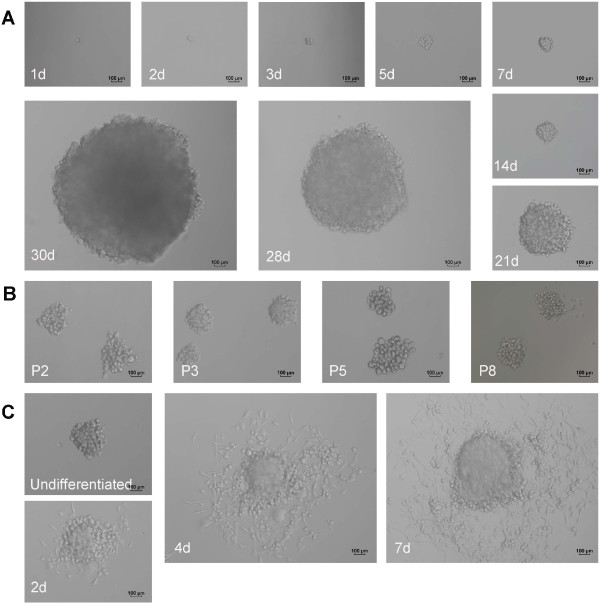
**Morphology of MCSCs under serum-free medium and medium containing 10% fetal bovine serum (FBS). (A)** Representative example of a CSC sphere formation originating from a single MB49 cell under an optimal serum-free culture medium. **(B)** MCSCs generated secondary passages. **(C)** MCSCs are differentiated and adherent to the culture dish when cultured in medium containing 10% FBS. (d = day; P = passage).

The limited dilution method showed that only a 2–3 percentage of MB49 cells generated CSC spheres in SFM. The passage 1 single MB49 cell formed a CSC sphere within 30 days in optimal SFM. The MCSCs were passaged after 15 days to form new tumor spheres, and most MCSCs generated secondary spheres. (Figure 2ab).

### Characterizations of MCSCs

#### Expression of CSCs markers

**Figure 3:**
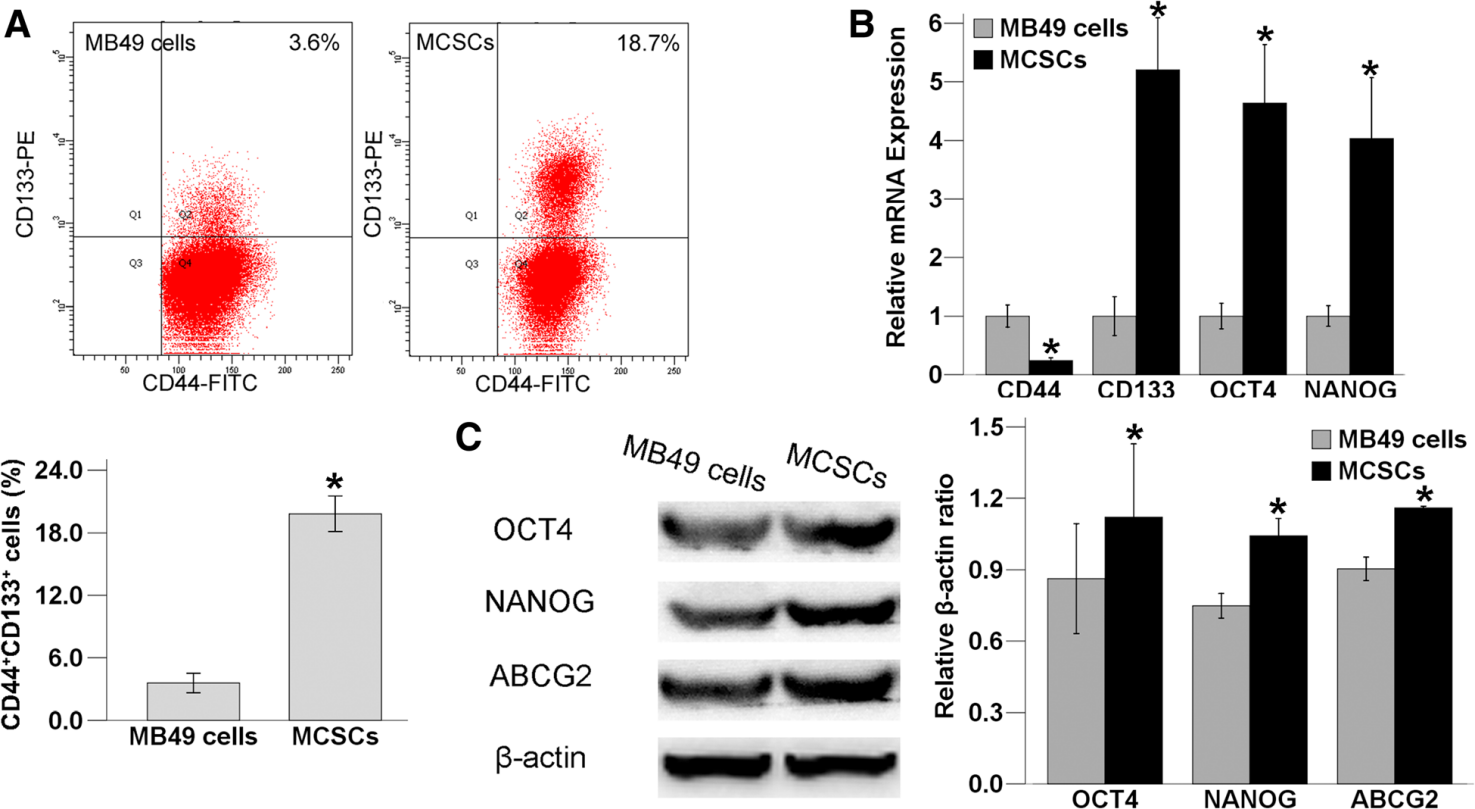
**Comparison of specific markers in MCSCs and MB49 cells. (A)** (up) In the fluorescence activated cell sorting analysis, the fraction of CD44^+^ cells in MB49 cells is higher. However, the fraction of CD44^+^CD133^+^ cells is higher in MCSCs than in MB49 cells. (down) The fraction of CD44^+^CD133^+^ cells is 19.83 ± 0.68% in MCSCs and 3.57 ± 0.38% in MB49 cells, which is elevated in MCSCs relative to MB49 cells. **(B)** The comparison of quantitative polymerase chain reaction analysis shows that the expression of CD133, CD44, OCT4 and NANOG are higher in MCSCs than in MB49 cells. **(C)** (left) The pattern of OCT4, NANOG and ABCG2 expression using western blot. β-actin is used as a positive control. (right) Comparison of western blot analysis shows that OCT4, NANOG and ABCG2 are sparsely distributed in MB49 cells but are abundantly expressed in MCSCs. (*P < 0.05).

As demonstrated by the FACS analysis, the fraction of CD44^+^ cells in MB49 cells was higher than that of MCSCs. However, the fraction of CD133^+^CD44^+^ cells was 19.83 ± 0.68% in MCSCs and 3.57 ± 0.38% in MB49 cells, which was elevated in MCSCs relative to MB49 cells (P < 0.05, Figure 3a).

The relative levels of CD133, OCT4 and NANOG were higher in MCSCs using the qPCR experiment, being 5 times as high as observed in MB49 cells. However, the level of CD44 was higher in MB49 cells (P < 0.05, Figure 3b).

OCT4, NANOG and ABCG2 were both expressed in MCSCs and in MB49 cells by the WB assay. They were sparsely distributed in MB49 cells, but they were abundantly expressed in MCSCs (P < 0.05,Figure 3c).

#### Differentiation

MCSCs were globular and floating in SFM. When the MCSCs were reseeded with a medium containing 10% FBS, they became flat after being differentiated and attached to the culture dish (Figure 2c).

#### Functional comparison

**Figure 4:**
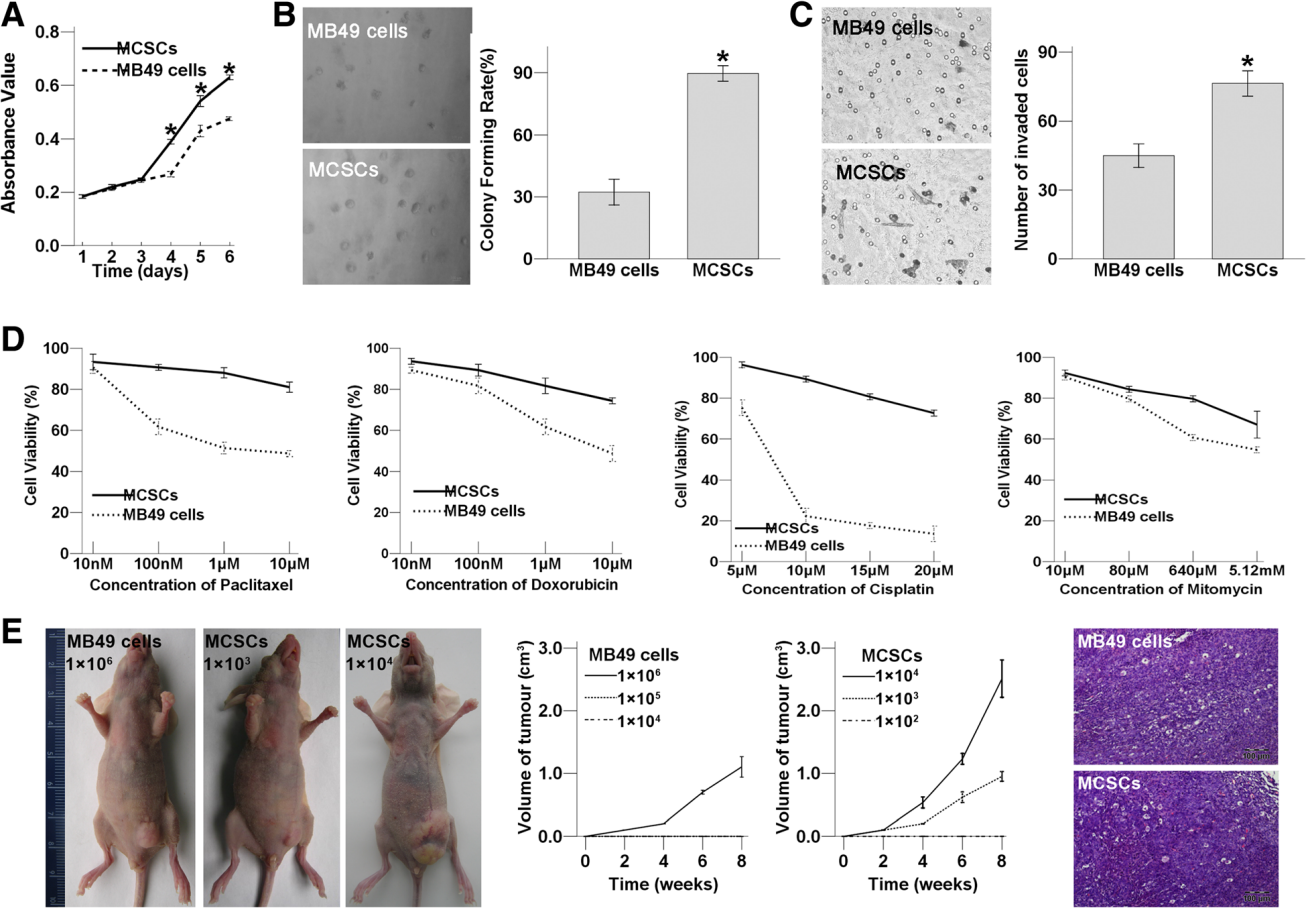
**Functional characteristics comparison in MCSCs and MB49 cells. (A)** Comparison of proliferative abilities. The cell proliferation growth curve using CCK-8 shows that MCSCs contain the higher absorbance value at day 4,5,6. **(B)** (left) The light images of soft agar assay shows that MCSCs form more numerous and bigger colonies than MB49 cells. (right) The comparison of colony number shows that MCSCs cause a significant increase in colony numbers on soft agar than MB49 cells do. **(C)** Comparison of migration abilities in vitro. (left) The light images of penetrating cells by transwell migration assays. (right) The numbers of invaded MCSCs are more than MB49 cells. **(D)** Comparison of resistance to chemotherapy using CCK-8. Compared to MB49 cells, MCSCs show higher cell viabilities after treatment with various concentrations of anti-cancer drugs including paclitaxel, doxorubicin, cisplatin and mitomycin. The viability is presented as% of viable cells after drug treatment relative to number of untreated control cells. Data is the mean ± SD of three independent experiments. **(E)** Comparison of tumorigenic abilities in vivo. (left) The light images of subcutaneously tumorigenicity by xenograft formation in deficient nude mice. (middle) MCSCs cause remarkable tumor volume than MB49 cells do following an injection of the same number. (right) Microphotographs of H&E stained tumor tissue sections from MCSCs and MB49 cells. (*P < 0.05).

MCSCs increased the proliferation as compared with MB49 cells in the SFM on day 4, 5, 6 after using CCK-8 in the cell proliferation assay (P < 0.05; Figure 4a). The soft agar assay revealed that MCSCs formed bigger and more numerous colonies than MB49 cells did (P < 0.05; Figure 4b). Both assays showed that MCSCs possessed highly proliferative abilities.

Under the same incubation conditions, the number of invaded MCSCs were more than that of MB49 cells (P < 0.05; Figure 4c). Transwell migration assays displayed that MCSCs contained higher transmembrane activity than MB49 cells.

Compared to MB49 cells, MCSCs showed higher cell viabilities after being treated with different concentrations of mitomycin, cisplatin, paclitaxel, and doxorubicin in Figure 4d. MCSCs demonstrated lower susceptibility to all these traditional anticancer agents.

MCSCs caused a more remarkable tumor volume than MB49 cells did. Immune deficient nude mice injected with 1 × 10^6^ in MB49 cells or 1 × 10^3^ in MCSCs formed xenografts, those injected with 1 × 10^5^ in MB49 cells or 1 × 10^2^ in MCSCs did not. The morphology of H&E stained xenograft tumor sections from MCSCs resembled tumor tissue from MB49 cells (Figure 4e). Xenograft formation showed that MCSCs possessed strongly tumorigenic ability in vivo.

## Discussion and conclusions

**Figure 5:**
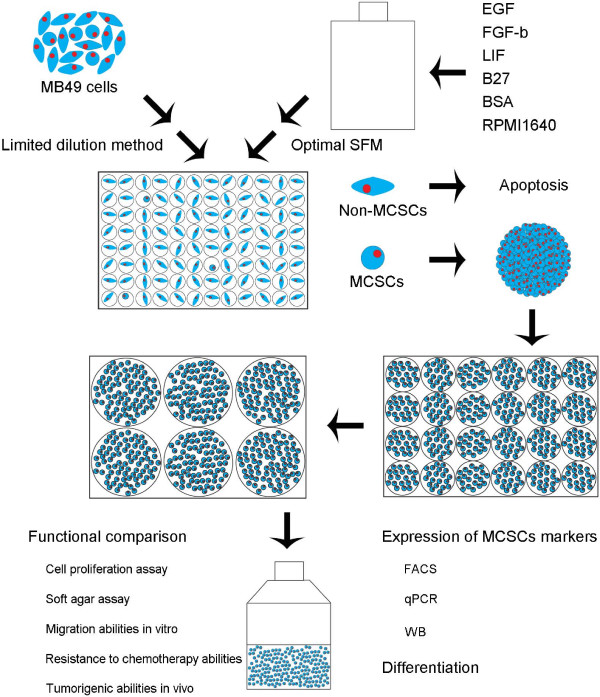
Diagram illustration to the proposed model for isolation of MCSCs by combination of limited dilution method and optimal SFM method.

To facilitate the transition of MCSCs to vaccine applications, advances in expanding MCSCs had become an absolute necessity. There were three methods that have been used to isolate CSCs from tumors: specific cell surface markers, SFM, and side population cells. These methods were limited due to the lack of purity of CSCs or the purity was not enough for CSCs [6]. Limited dilution assay was mostly used to assess the tumorigenic activity of xenograft cells [15]. However, the limited dilution method in our study was different, which allowed MCSCs sphere formation to originate from a single cell before improving the purity of CSCs (Figure 5). The limited dilution method showed that only a small percentage of MB49 cells generated CSC spheres, and the proportion was similar to other scholars’ results [16].

Considering that the serum caused irreversible differentiation of stem cells, SFM selection might be useful for CSCs expansion and would allow for maintenance of an undifferentiated stem cell status [17]. Accutase, which is one kind of digested enzyme, was different from trypsin and did not need serum to stop digestion. Accutase stopped digestion by adding 50 times as much SFM to dilute the enzyme, so MCSCs could maintain an undifferentiated status.

Due to the heterogeneity of different cancers, the optimal medium for the growth of CSCs may vary from case to case. A supplement medium that is critical to one line may be of no benefit or even adverse to the other. Therefore, it is recommended to perform experiments by choosing the optimal supplement composition for the MB49 cell line. Our study suggests that the medium should contain four stimulated factors: LIF, B27, EGF and FGF-b, which would together provide an optimal sphere formation with the MCSCs having survived continuous passage in culture. In contrast, MB49 cells do not proliferate and expire under SFM during serial passages. To our knowledge, this is the first report about the isolation and expansion of MCSCs via a combination of limited dilution and SFM methods, which is modified to improve the purity of CSCs.

CD133 and CD44 have been used to identify CSCs from other cancer tissues [18,19]. Interestingly, our results showed an elevated CD44^+^CD133^+^ expression in MCSCs. OCT4 played a significant role in self-renewal [20], and NANOG was identified as a key molecule to maintain self-renewal and to block differentiation [21]. These genes can potentially lead to tumorigenesis and affect some cancer behaviors, such as resistance to therapies or cancer recurrence[6]. They were not only upregulated at the protein level (WB) but also at the mRNA transcript level (qPCR) in MCSCs.

The capability to differentiate was another important feature of CSCs [8]. Furthermore, we applied the techniques to functionally characterize MCSCs populations[22,23]. MCSCs had typical CSCs that were capable of self-regeneration with a higher proliferative capacity and greater colony formation potential. MCSCs showed the greater capacity to penetrate wells, which indicated that these cells were the most likely to migrate.

Chemotherapy killed the majority of cells in a tumor, but it did not kill CSCs, which might be the mechanism behind the resistance to chemotherapy [24,25]. MCSCs had a lower susceptibility to mitomycin, cisplatin, paclitaxel and doxorubicin. The ATP-binding cassette (ABC) transporters explained the mechanism that many chemical drugs were pumped out of cells by ABC transporters [26]. MCSCs showed a higher level of ABCG2 expression at the protein level (WB), and the upregulation of ABCG2 was associated with the resistance of MCSCs to anti-cancer drugs. The standard experimental method for the isolation of CSCs was to test the tumorigenicity of cancer cells in immunodeficient mice [27]. MCSCs showed the greatest ability to form tumors in the subcutaneous tissues of immunodeficient mice.

Taken together, this data showed that cultured MCSCs displayed specific CSC properties. In conclusion, MCSCs were isolated successfully with a modified method using a combination of limited dilution and SFM methods. MCSCs contained characteristics resembling CSCs such as in vitro self-renewal, a differentiation potential, chemotherapy resistance and in vivo tumorigenic capacity. MCSCs may provide an ideal model for the development of bladder cancer vaccine research.

## Competing interest

All authors have no conflict of interest regarding this paper.

## Author contributions

YZ, YD and WT conceived and designed the experiments; YZ, CL, NL, YL and CH performed the experiments; YZ, WC and FL analyzed the data; YZ, NL, WC and FL contributed reagents/materials/analysis tools; YZ, NL and WT wrote the paper; All authors read and approved the final manuscript.

## Pre-publication history

The pre-publication history for this paper can be accessed here:

http://www.biomedcentral.com/1471-2490/13/57/prepub
